# Biomimetic Active Stereo Camera System with Variable FOV

**DOI:** 10.3390/biomimetics9120740

**Published:** 2024-12-04

**Authors:** Yanmiao Zhou, Xin Wang

**Affiliations:** School of Mechanical Engineering and Automation, Harbin Institute of Technology Shenzhen, Shenzhen 518055, China

**Keywords:** bionic eye, active vision, dynamic calibration, variable FOV

## Abstract

Inspired by the biological eye movements of fish such as pipefish and sandlances, this paper presents a novel dynamic calibration method specifically for active stereo vision systems to address the challenges of active cameras with varying fields of view (FOVs). By integrating static calibration based on camera rotation angles with dynamic updates of extrinsic parameters, the method leverages relative pose adjustments between the rotation axis and cameras to update extrinsic parameters continuously in real-time. It facilitates epipolar rectification as the FOV changes, and enables precise disparity computation and accurate depth information acquisition. Based on the dynamic calibration method, we develop a two-DOF bionic active camera system including two cameras driven by motors to mimic the movement of biological eyes; this compact system has a large range of visual data. Experimental results show that the calibration method is effective, and achieves high accuracy in extrinsic parameter calculations during FOV adjustments.

## 1. Introduction

In recent years, with the improvement of robotics and autonomous driving technologies, visual perception is playing an increasingly important role in various fields [1]. In the field of autonomous driving, visual sensing systems provide vehicles with real-time and accurate environmental information; through image analysis, they efficiently detect and recognize various elements such as roads, obstacles, and pedestrians, thereby enabling safe navigation and path planning [2,3]. Similarly, in other fields, visual perception not only improves the autonomy of robots, but also expands their adaptability in complex scenarios. Especially in dynamic and unknown environments, visual systems can obtain rich three-dimensional information through image data, providing robots with higher decision-making and positioning accuracy, and those advantages made robots more widely used in manufacturing, services, medical care, agriculture, and other fields [4,5,6], and have greatly promoted the process of intelligence and automation.

Currently, vision systems have become a crucial component in robotic sensing systems [7] due to their advantages such as their low cost and high accuracy. In contrast to monocular systems, binocular vision systems can utilize the principle of parallax to extract th depth information of objects from two images [8], thus acquiring three-dimensional information about the scene for high-precision 3D reconstruction, which is crucial for tasks such as precise positioning and obstacle avoidance in robots. It also provides higher measurement accuracy, exhibits greater robustness to lighting changes and noise, and can operate stably in more complex environments, thereby further enhancing the operational capabilities of robots across various domains [9].

In addition to traditional binocular systems with fixed-angle installation, many researchers have developed active binocular vision systems that mimic the movement patterns of the human eye, enabling robots to perceive their environment in a manner that is similar to human vision. For instance, the Artificial Intelligence Laboratory at MIT developed the Kismet robot [10], which has three degrees of freedom to control its visual direction, allowing it to move and adjust its gaze across different areas. Cannata [11] of the University of Genoa created a robotic bionic eye and proposed an eye movement model along with a muscle-movement algorithm to simulate the actual scanning and smooth tracking motions of the human eye. However, both the Kismet robot and Cannata’s bionic eye have a limitation: the left and right eyes always move in the same direction, meaning that the overall FOV of the camera remains unchanged. In addition to imitating human eyes, many researchers have drawn inspiration from animals and have developed various bionic vision systems based on the eye structures of different species. For example, Ru [12] was inspired by the bionic arrangement of fish eyes and designed a bionic fisheye camera. However, to achieve a larger FOV, they employed a fisheye camera, which resulted in significant image distortion and small overlap area. Similarly, Bae [13] designed a stereoscopic artificial compound-eye system that mimics the structure of insect compound eyes. This design enables the system to efficiently capture depth information and adapt to variations in dynamic scenes, but similar to traditional compound-eye designs, it faces a limited FOV overlap area.

To enable binocular cameras to adjust their FOV angle and flexibly change perspective, many researchers have proposed systems with independent rotation for the left and right cameras. For instance, Wang [14] developed an active binocular camera with four degrees of freedom, where each camera allows independent pitch and opening rotations, and they established a coordinate system at the center of the camera baselines, deriving depth information through the geometric relationship of relative object points in this coordinate system. Their approach simplifies calculations, but it requires the imaging plane to be parallel in the camera’s initial state, and the camera’s rotation center should coincide with the origin of its coordinate system to accurately compute the image point position after rotation. Zhang [15] designed a bionic binocular camera, featuring two degrees of freedom for each camera, allowing rotation around both horizontal and vertical axes and thus expanding the camera’s FOV significantly; he proposed two models for binocular cameras: one scenario involves the rotation of only the camera, while another scenario considers the simultaneous rotation of both the camera and its axis. Compared to other methods, this design simplifies installation as it does not require the camera coordinate system’s position or rotation axis alignment. However, left and right camera rotation is still consistent, which limits the FOV and depth-detection range. Xu [16] developed an active stereo vision system where two cameras are connected by a synchronous belt to two axes. This configuration enables the cameras to rotate in opposite directions by equal angles from their shared centerline. The camera model she proposed allows a certain offset between the left camera and the rotation axis, which places certain restrictions on the design of the camera.

In contrast to the conventional fixed stereo vision system, the active stereo vision system can dynamically adjust the extrinsic parameters of the cameras [17]. However, any changes in these parameters require re-calibration, significantly increasing the workload. Consequently, many researchers have focused on developing calibration methods specifically for active stereo camera systems. Kwon [18] proposed a calibration method for active stereo systems that explicitly estimates the position and orientation of the cameras’ rotation axes. This approach enhances adaptability to large translational and angular changes. Mohamed [19] proposed an online geometric correction method based on motor encoder angles. This method establishes a linear mapping between motor angles and image angles through offline calibration and uses this mapping to update the essential matrix in real-time. The existing methods are either only for offline calibration or only for fitting the relationship between parameters and angles, and their accuracy is limited by the sensor.

Unlike humans, certain fish species, such as pipefish and sandlances, have the remarkable ability to control their eyes independently. The eye muscles of these fish function separately, allowing each eye to rotate independently [20]. This flexibility enables them to observe different directions simultaneously, maximizing their FOV. Inspired by this special eye control method and considering the current state of research in active vision systems, we propose a dynamic calibration method for active stereo cameras. Unlike other calibration methods, this approach imposes no specific requirements on the camera installation position, thereby eliminating the need to measure the relative positions of the camera’s optical center and the rotation axis. Static parameter calibration is achieved solely through images of a calibration board captured before and after camera rotation, allowing for real-time parameter updates as the camera angles change. Based on this method, a bio-inspired active stereo camera system with a variable FOV is designed. Unlike conventional visual systems, this camera employs gears and housing designs to facilitate adjustments in the angle between the cameras and the overall pitch angle as needed, resulting in a more compact size compared to existing active camera systems, thus broadening its application potential.

The contributions of this paper include three main aspects: First, an algorithm for updating the relative extrinsic parameter matrix of the active stereo camera based on camera rotation angles is proposed in response to changes in the field of view. Second, in conjunction with the extrinsic parameter matrix update algorithm, a bio-inspired active stereo vision system with a variable FOV is designed and implemented, featuring biologically inspired changes in visual range that allow for adjustments to the FOV as required. Finally, dynamic stereo camera calibration experiments are conducted, demonstrating that the proposed algorithm for updating the relative extrinsic parameter matrix achieves a high level of accuracy.

The remainder of this paper is organized as follows: Section 2 presents an algorithm for static camera parameter calibration and dynamic relative pose updates based on camera rotation angles, primarily applied to stereo camera calibration. Section 3 describes the design of a bio-inspired active stereo camera system with two degrees of freedom, based on the proposed calibration method. In Section 4, dynamic calibration measurement experiments are conducted using this active bio-inspired stereo camera system. Finally, Section 5 provides a summary of this study.

## 2. Rotation Angle-Based Calibration of Binocular Camera

Depth extraction is a fundamental basis for most tasks carried out by various vision systems [21]. To achieve precise depth extraction, the calibration of the binocular camera is necessary after installation. The internal parameters of calibration describe the inherent characteristics of the camera imaging system, including focal length, principal point, and distortion coefficients. These parameters are essential for correcting image distortion caused by the camera lens. The extrinsic parameters describe the relative position and orientation of the camera with respect to the world coordinate system, typically represented as a rotation matrix and a translation vector. The extrinsic parameters are used to map 2D image points to their actual positions in 3D space. The objective of extrinsic calibration for binocular cameras is to obtain the relative pose relationship between the two cameras, enabling the depth information extraction of objects in the scene through the disparity method, which is a critical step in stereo vision 3D reconstruction.

For standard binocular cameras, the most commonly used calibration method is Zhang’s calibration method [22]. This method is based on a 2D planar pattern and involves capturing images of a calibration board from different angles. By extracting the coordinates of the corner points of the chessboard pattern in the images and mapping these to the known 3D coordinates in the world coordinate system, a linear method is used for preliminary estimation, resulting in the internal and extrinsic parameters of the camera. For binocular camera calibration, in addition to performing intrinsic calibration for each camera individually, it is necessary to estimate the relative pose, i.e., the extrinsic parameters of the binocular calibration. Specifically, the calibration process begins with independent intrinsic calibration for both cameras, followed by simultaneous image capture of the same calibration board. The corner point information for the same feature point is extracted in the coordinate systems of both cameras, allowing the calculation of the rotation matrix and translation vector between the two cameras based on the principle of triangulation.

However, the results of ordinary binocular calibration are only valid for the fixed relative position of the cameras when capturing the calibration board. If the relative position changes, the calibration results become meaningless. Continuing to use these outdated results significantly affects the accuracy of the disparity map and other visual information collected by the binocular cameras. Therefore, for binocular systems where the relative position frequently changes, it is necessary to recalibrate the cameras each time a positional change occurs. This recalibration entails re-acquiring images of the calibration board and recalculating the internal and extrinsic parameters, which is cumbersome for robots equipped with binocular cameras.

To address this issue, enabling the cameras to complete calibration tasks concurrently with pose changes, this paper proposes an algorithm for static camera parameter calibration and dynamic relative pose updates based on camera rotation angles. The general approach is as follows: Ihe ultimate goal of binocular calibration is to obtain the relative position of the two cameras, specifically the rotation R and translation T matrices of one camera relative to the other. In the initial fixed position, we can utilize Zhang’s calibration method to accurately obtain the extrinsic parameters. Therefore, we first fix the binocular cameras in parallel, treating this position as the initial state, and use Zhang’s method to derive the extrinsic parameter matrix in this parallel configuration. When the driving motors alter the camera angles, we record the angle values relative to the initial position. With the known change in angles, we can calculate the new R and T matrices using the initial R and T matrices and the rotation angles, thus completing the calibration for the binocular system post-rotation.

First, we compute the extrinsic parameters of the binocular cameras in the initial position. With the binocular cameras arranged in parallel, we use chessboard images as calibration boards and capture images from different angles. Initially, intrinsic calibration is performed to obtain the internal and extrinsic parameters of each camera. Then, we analyze the coordinates of the same feature point in both camera coordinate systems, leading to the following relationships:

(1) $$\left\{\begin{array}{cc} P_{l} & =R_{l}\cdot P_{w}+T_{l}\\ P_{r} & =R_{r}\cdot P_{w}+T_{r} \end{array},\right.$$

By transforming these equations, we can express $P_{w}$ in both coordinate systems:

(2) $$\left\{\begin{array}{cc} {R_{l}}^{-1}P_{l}-T_{l} & =P_{w}\\ {R_{r}}^{-1}P_{r}-T_{r} & =P_{w} \end{array},\right.$$

Subtracting the two equations gives:

(3) $${R_{l}}^{-1}P_{l}-T_{l}-{R_{r}}^{-1}P_{r}-T_{r}=0,$$

Multiplying by $R_{r}$ and simplifying yields:

(4) $$P_{r}=\left({R_{r}R_{l}}^{-1}\right)P_{l}+\left(T_{r}-{R_{r}R_{l}}^{-1}T_{l}\right),$$

From this, we derive:

(5) $$\left\{\begin{array}{c} P_{r}=R\cdot P_{l}+T_{R}\\ T=T_{r}-{R_{r}R_{l}}^{-1}T_{l} \end{array}.\right.$$

Finally, we obtain the pose relationship of the right camera relative to the left camera, represented by the rotation R and translation T matrices. Since the cameras are arranged in parallel, this configuration is referred to as the initial state, denoted as $R_{0}$ and $T_{0}$. If the camera angles remain unchanged, the computed $R_{0}$ and $T_{0}$ matrices can be used for the rectification and stereo matching of the left and right cameras, producing disparity images under the condition of parallel camera arrangement.

### 2.1. Active Camera Model

Next, we consider the rotation of the cameras. During the adjustment of pitch angle, the entire binocular camera can be rotated, while the relative position between the left and right cameras remains unchanged. Therefore, this paper only considers the change in the extrinsic parameters caused by the adjustment of the opening angle.

**Figure 1:**
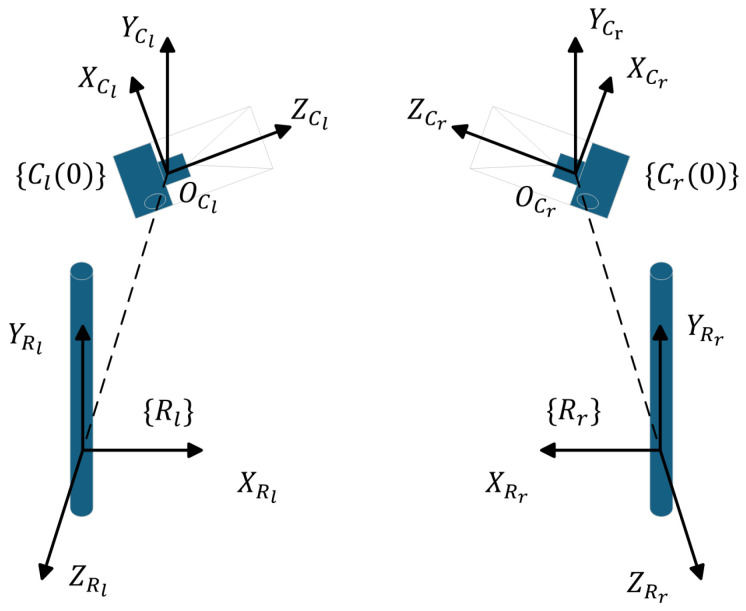
$R_{l}$ represents the coordinate system of the left camera’s rotation axis, while $R_{r}$ represents the coordinate system of the right camera’s rotation axis. $C_{l}\left(0\right)$ refers to the coordinate system of the left camera at the initial state (θ=0), and $C_{l}\left(\theta \right)$ refers to the left camera’s coordinate system after rotation by an angle θ. Similarly, $C_{r}\left(0\right)$ refers to the coordinate system of the right camera at the initial state (θ=0), and $C_{r}\left(\theta \right)$ refers to the right camera’s coordinate system after rotation by an angle θ.

Due to the unique structure of the rotation mechanism, the rotation axis remains fixed during the adjustment of the opening angle. Consequently, the rotation axis coordinate system stays constant throughout the camera’s rotation. In the initial state, we establish a coordinate system at the optical centers of both cameras and at the rotation axis, referred to as the camera coordinate system and the rotation axis coordinate system. The origin of the rotation axis coordinate system can theoretically be any point along the rotation axis; however, for ease of computation, we take the intersection of the xz-plane of the camera coordinate system with the rotation axis as the origin of the rotation axis coordinate system. The z axis of the rotation axis coordinate system points from the origin of the camera coordinate system to the origin of the rotation axis coordinate system, while the directions of the other two axes remain aligned with those of the camera coordinate system. In the world coordinate system, their representations are denoted as $\left\{R_{l}\right\}$, $\left\{R_{r}\right\}$, $\left\{C_{l}\left(\theta \right)\right\}$, and $\left\{C_{r}\left(\theta \right)\right\}$. The coordinate system diagram is shown in Figure 1.

We assume that the transformation matrices from the camera coordinate systems to the rotation axis coordinate system are represented as TClRl(θ) and TCrRr(θ). In the initial state, these transformation matrices are defined as TClRl(0) and TCrRr(0). Using these transformation matrices, we can map the camera coordinate systems from their initial positions to the rotation axis coordinate system:

(6) ClRl(0)=TClRl(0)Cl(0)CrRr(0)=TCrRr(0)Cr(0).

After a rotation by a certain angle θ, the cameras move from their initial positions to new positions, represented by $\left\{C_{l}\left(\theta \right)\right\}$ and $\left\{C_{r}\left(\theta \right)\right\}$. Since the rotation is around a fixed axis, we can use the rotation matrix of the y axis, Rot(y,θ), to express the rotated camera coordinate systems in the rotation axis coordinate system:

(7) ClRl(θ)=Rot(y,θ)TClRl(0)Cl(0)CrRr(θ)=Rot(y,θ)TCrRr(0)Cr(0).

Since the initial poses and the rotation axis coordinate system remain unchanged, the values of TClRl(0)Cl(0) and TCrRr(0)Cr(0) are constant. We refer to these as the initial pose matrices of the cameras, denoted as $T_{l}$ and $T_{r}$. The rotated camera coordinate systems in the rotation axis frame, ClRl(θ) and CrRr(θ), are represented by the transformation matrices TClRl(θ) and TCrRr(θ). Therefore, the above equations can be simplified as follows:

(8) TClRl(θ)=Rot(y,θ)TlTCrRr(θ)=Rot(y,θ)Tr.

The ultimate goal of binocular calibration is to obtain the relative pose matrix between the two cameras, denoted as TCrCl(θ). By introducing the rotation axis coordinate system, which remains stationary, we can first determine the relative poses of the left and right cameras concerning the rotation axis. Using these relative poses, we can calculate the relative pose matrix between the two cameras as follows:

(9) TCrCl(θ)=TRlCl(θ)·TRrRl·TCrRr(θ),

By combining this with the expression for the rotated camera position, we get:

(10) TCrCl(θ)=(Rot(y,θ)Tl)−1·TRrRl·Rot(y,θ)Tr.

Here, TRrRl represents the relative pose matrix between the left and right rotation axes. Using Zhang’s calibration method, we can determine the relative position between the two cameras for any angle TCrCl(θ). Taking the relative pose at the initial position, TCrCl(0), we can derive the relative pose matrix between the left and right rotation axes as follows:

(11) TRrRl=TClRl·TCrCl(0)·TRrCr=Tl·TCrCl(0)·Tr.

To minimize the error in TRrRl, multiple angles can be used to calculate and average the results. The final remaining components to solve are the initial pose matrices $T_{r}$ and $T_{l}$ of the cameras.

### 2.2. Solving of Initial Pose Matirx

**Figure 2:**
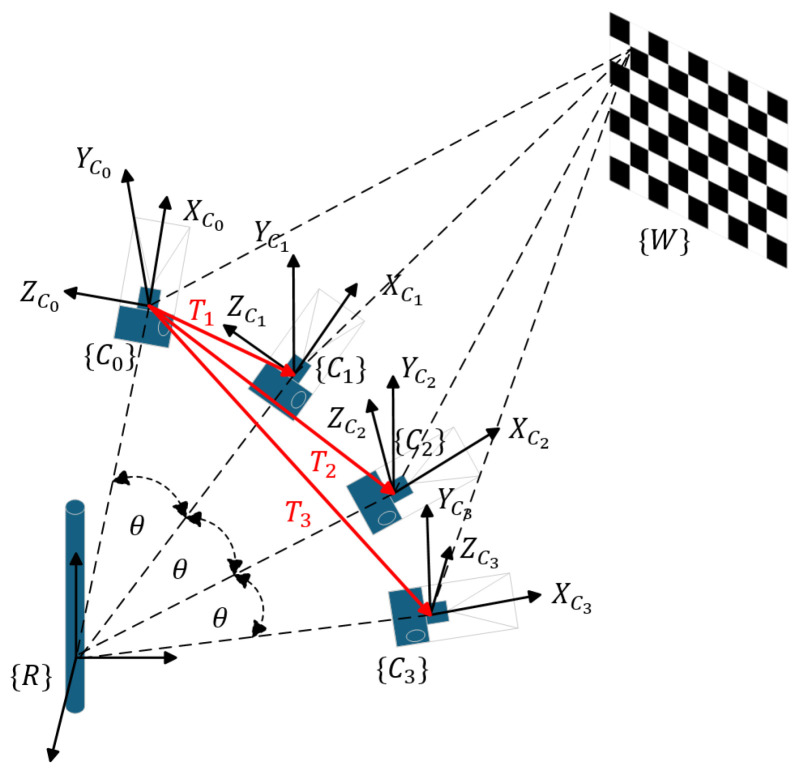
This diagram shows the position of the camera after rotating by an angle θ relative to the rotation axis coordinate system, and $T_{i}$ represents the vector from the initial position of the camera’s optical center to the position of the camera’s optical center after *i* rotations.

First, we apply Zhang’s calibration method to obtain the intrinsic matrices of the left and right cameras. Starting from the initial position, the cameras are rotated incrementally by specific angles, with the calibration board remaining fixed throughout the process. Since the same calibration board is used at each position, and calibration occurs in the same location, the world coordinate system {W} remains unchanged across multiple calibration angles. The relative camera poses at different angles are shown in Figure 2.

Through single calibration at each position, we obtain the pose transformation matrices of the camera coordinate system relative to the calibration board at each position, denoted as TiW. Using these transformation matrices, we extract the translation component to find the coordinates of the camera coordinate system origin in the world coordinate system for each position. By using the initial position as a reference, we subtract the translation vector of the camera origin at the initial position from each position’s translation vector, resulting in a set of direction vectors $T_{i}$ originating from the initial position. These vectors lie on the rotation plane and are orthogonal to the rotation axis; thus, the cross-product of any two vectors, $y_{\mathrm{cross}}$, provides the direction of the rotation axis vector y.

Since the vector origin is at the initial position’s camera coordinate system, this direction is expressed in the initial camera coordinate system as the unit vector along the y axis of the rotation axis in the initial camera coordinate system, denoted by yRC0. To reduce errors, we calculate the cross-products of each pair of vectors and then take the average, yielding the optimal result:

(12) yRC0=∑(Ti×Tj)/|Ti×Tj|n.

Next, we connect the origin of the rotation axis coordinate system to the origins of the camera coordinate systems at each position. Since the *x* axis of the rotation axis coordinate system is aligned with the line connecting the origin of the rotation axis coordinate system and the initial camera position, and since we already know the direction vectors $T_{i}$ from the initial camera position to each position, along with the angles between each camera origin and the rotation axis origin, we can calculate the radius *r* of rotation as follows:

(13) $$r=\frac{\sum \frac{|T_{i}|/2}{\sin(\theta /2)}}{n}.$$

With the rotation axis vector yRC0 determined from the previous step, the Rodrigues’ rotation formula provides the rotation matrix R about this axis. For instance, rotating $T_{1}$ counterclockwise by $(180^{\circ }-\theta )/2$ and normalizing it yields the *x* axis direction vector of the rotation axis coordinate system. Repeating this for all $T_{i}$ and averaging results yields the optimal direction of the rotation axis coordinate system’s *x* axis in the initial camera coordinate system, denoted as xRC0:

(14) xRC0=∑Rodrigues(y,θ)·Ti/|Rodrigues(y,θ)·Ti|n.

Then, by taking the cross-product of the *x* and *y* axis unit vectors, we obtain the z axis unit vector of the rotation axis coordinate system in the initial camera coordinate system, denoted as zRC0:

(15) zRC0=yRC0×xRC0.

Finally, by multiplying the *x* axis unit vector of the rotation axis coordinate system by the rotation radius *r*, we obtain the translation vector t from the initial camera position to the rotation axis coordinate system:

(16) t=r·xRC0.

Using the parameters obtained above, we describe the rotation axis coordinate system in the initial camera coordinate system as TRC0(0):

(17) TRC0(0)=(xRC0)T(yRC0)T(zRC0)TtT0001.

From this matrix, we obtain the initial pose matrix T as:

(18) T=TCR(0)=TRC(0)−1.

Finally, substituting this matrix into the following expression:

(19) TCrCl(θ)=Rot(y,θ)·Tl−1·TRrRl·Rot(y,θ)·Tr.

We can compute the relative pose matrix between the left and right cameras at any position, enabling disparity calculation and depth information retrieval.

## 3. Variable FOV Binocular Vision System

**Figure 3:**
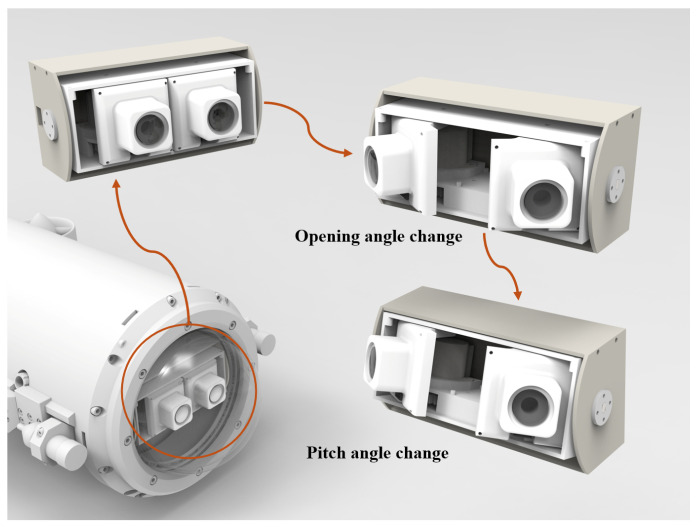
Active binocular vision system, illustrating its application scenario as well as the sequential process of opening, closing, and performing pitch rotations.

The primary research objective of the active stereo camera system with a variable FOV is to mimic the different motion states of biological eyes under varying conditions [23]. This allows for dynamic changes in both the camera’s visual range and depth measurement capabilities. When a wide FOV is required, the camera’s field angle is expanded; conversely, when more depth information is needed, the field angle is reduced to increase the overlap between the two cameras’ FOVs, thereby enhancing the depth measurement range. To meet this requirement, this study employs motors to drive the camera rotation, facilitating changes in the FOV. We designed a bio-inspired active stereo vision system with a variable FOV, as illustrated in Figure 3.

### 3.1. System Structure and Design

In common robotics, the visual components are often mounted in a fixed position on the robot body [24], with the camera’s FOV relying on the robot’s own movement. To achieve the design goal of an adjustable visibility range and depth measurement range according to task requirements, an angle-adjustable base needs to be designed and fixed to the robot body. Considering the limited internal space of the robot, which does not allow for much room for the vision system, it is essential to meet the installation requirements for compact spaces. Additionally, to facilitate later maintenance, a mechanism that is as simple as possible should be employed to adjust the opening and closing angles while keeping the overall dimensions of the camera as small as possible.

**Figure 4:**
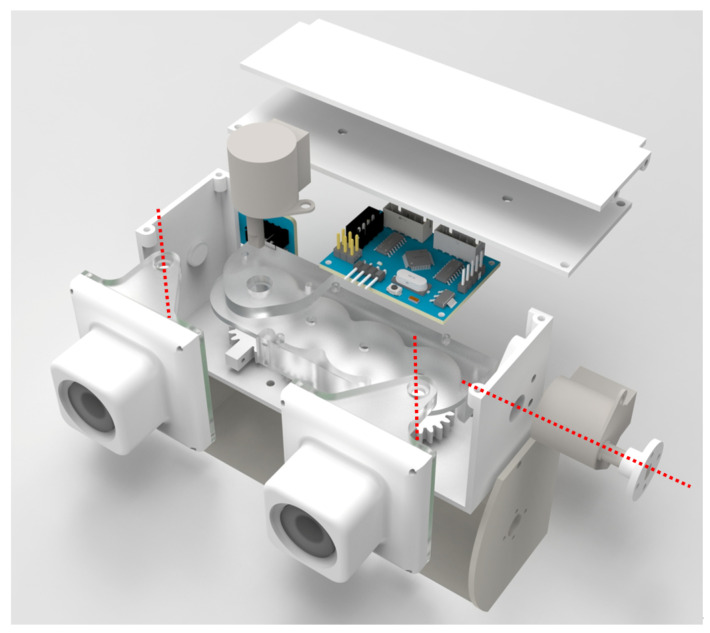
Schematic diagram of the overall structure; the red dotted line in the figure is the rotation axis.

Given that the binocular camera has a certain distance between the optical axes due to the baseline, and considering the requirements for overall size control and stability during camera rotation, a multi-stage gear transmission is used to achieve symmetrical changes in the opening and closing angles of the binocular camera. The overall structure is shown in Figure 4, consisting of a motor, four gears with the same number of teeth, an outer shell, and camera connectors. The motor drives gear 1, which, through the multi-stage gear system, enables the two cameras rigidly connected to gears 1 and 4 to rotate symmetrically, thereby fulfilling the design requirements for horizontal changes in the FOV.

In designing the mechanism for adjusting the FOV in the pitch direction, it is essential to ensure that the optical centers of the two cameras are positioned on the same horizontal plane to meet the accuracy requirements for depth acquisition. Therefore, both the left and right cameras must rotate simultaneously while maintaining a consistent angle. To achieve this, the platform is considered as a whole for synchronized rotation. Consequently, the outer casing of the stereo camera is designed in an inner and outer shell structure, consisting of a motor, an inner shell, an outer shell, and connecting components. When the outer shell is fixed to the body of the robot, the motor drives the inner shell to rotate relative to the outer shell, fulfilling the design requirements for adjusting the pitch angle of the FOV.

After the comprehensive consideration of the dimensions and various performance parameters of the motor, we chose a small stepper motor as the drive motor as the drive motor. For the main control, the STM32C8T6 from STMicroelectronics (Quakertown, PA, USA) is chosen as the main control chip to control the motor rotation and communicate with the upper computer, meeting the design requirements of the camera with its performance and compact size.

### 3.2. Camera Performance Parameters

**Figure 5:**
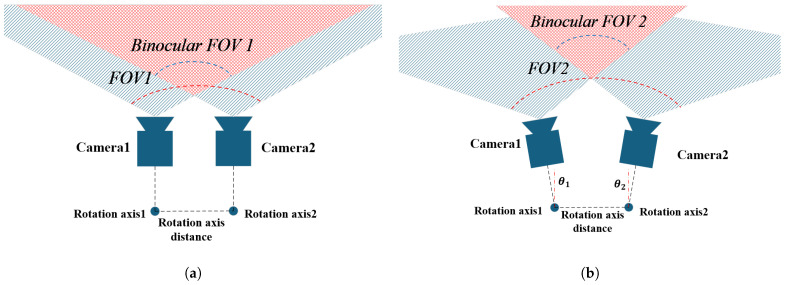
Camera FOV diagram. (**a**) shows the normal FOV of the camera. (**b**) shows the FOV when the camera’s aperture angle is increased.The blue cone in front of the camera represents the FOV of the single camera, and the red cone represents the common FOV of the two cameras, where depth can be obtained.

The camera model used is the IMX179 8MP USB Camera (A) from Waveshare Electronics (Shenzhen, China). The maximum image resolution achievable by the monocular camera is 3288 × 2512, with a horizontal FOV of $145^{\circ }$, a vertical FOV of $108^{\circ }$, and a focal length of 2.8 mm. After fixing it to the designed base, the overall FOV and depth acquisition area when arranged horizontally are shown in Figure 5a. When a wide FOV is required, the drive motor rotates the camera outward, and at the maximum angle, the overall FOV and depth acquisition area is shown in Figure 5b. The relevant parameters of the camera are shown in Table 1.

## 4. System Experiment and Testing

### Dynamic Calibration Experiment

To validate the dynamic relative pose matrix update method based on camera rotation angles, we conducted an experiment for updating the relative pose parameters using the constructed active stereo camera system. The dynamic calibration of the stereo camera aims to acquire the relative position between the two cameras. For this experiment, a checkerboard pattern with dimensions of 8 × 11 and a square size of 30 mm was used as the calibration board.

Initially, we precisely calibrated the intrinsic parameters of both cameras using the checkerboard pattern through OpenCV, achieving a reprojection error of less than 0.1 pixels. The intrinsic parameter matrices of the left and right cameras are expressed as follows:

(20) $$M_{l}=\left[\begin{array}{ccc} 827.35 & 0 & 646.49\\ 0 & 828.40 & 363.22\\ 0 & 0 & 1 \end{array}\right]$$

(21) $$M_{r}=\left[\begin{array}{ccc} 824.31 & 0 & 642.23\\ 0 & 825.69 & 403.31\\ 0 & 0 & 1 \end{array}\right]$$

The camera distortion coefficients are denoted as $k_{l}$ and $k_{r}$, representing the lens distortion coefficients for cameras $C_{l}$ and $C_{r}$, respectively. The distortion coefficients are expressed as follows:

(22) $$k_{l}=\left[\begin{array}{ccccc} -0.40756 & 0.22101 & 0.0010756 & -0.0011212 & -0.07115 \end{array}\right]$$

(23) $$k_{r}=\left[\begin{array}{ccccc} -0.39846 & 0.19864 & 0.0010451 & -0.00038817 & -0.05496 \end{array}\right]$$

**Figure 6:**
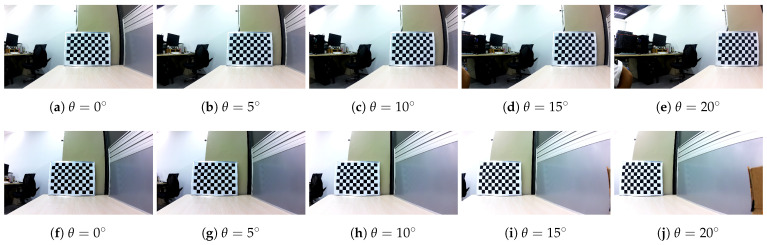
Camera calibration pictures. (**a**–**e**) are pictures taken when the left camera rotates from 0 degrees to 20 degrees, with each rotation of 5 degrees, (**f**–**j**) are pictures taken by right camera, also rotate 5 degrees each time, from 0 degrees to 20 degrees.

Furthermore, to obtain the relative pose relationship between the same camera at multiple rotations, we fixed the calibration board, ensuring that the acquired camera poses were always referenced to the same world coordinate system. Starting from the initial position, images of the calibration board were captured at different orientations. After each image capture, the opening angles of the left and right cameras were incrementally increased by $5^{\circ }$, resulting in a series of images as shown in Figure 6.

To determine the rotation matrix and translation vector of the camera coordinate system relative to the world coordinate system at various positions, we can treat this as a Perspective-n-Point (PnP) problem, given that the camera’s intrinsic parameters and distortion coefficients are known. A PnP-based method is employed to calculate the camera’s pose and convert it into the form of a homogeneous transformation matrix. After obtaining the camera’s pose at each position relative to the world coordinate system, we use the initial position as a reference point and subtract the pose of the initial position from the subsequent poses. This results in direction vectors $T_{i}$ originating from the origin of the camera coordinate system at the initial position. The final measurement results are presented in Table 2.

At θ=0, representing the initial position, the algorithm described in Section 2.2 processes the obtained translation vector to determine the representation of the rotation axis coordinates—specifically the *x*, *y*, and *z* axes—in the camera coordinate system. Additionally, it computes the translation vector t from the camera coordinate system to the rotation axis coordinate system. Converting these into a homogeneous transformation matrix yields the initial pose matrices $T_{r}$ and $T_{l}$:

(24) $$T_{r}=\left[\begin{array}{cccc} -0.26261 & -0.0064008 & -0.96512 & -29.07\\ 0.012359 & 1.0001 & 0.012225 & 0.019943\\ -0.9649 & 0.0087152 & 0.26249 & 0.042333\\ 0 & 0 & 0 & 1 \end{array}\right]$$

(25) $$T_{l}=\left[\begin{array}{cccc} 0.010823 & -0.0054781 & -0.99994 & -36.312\\ 0.0022947 & -0.99995 & 0.010946 & 0.014669\\ 0.99994 & 0.002413 & 0.01081 & 0.036864\\ 0 & 0 & 0 & 1 \end{array}\right]$$

In the images captured at θ=0, we performed stereo calibration to obtain the relative pose matrix TCrCl(0) of the left and right cameras. Based on the initial pose matrices $T_{r}$ and $T_{l}$, the relative pose matrix TRrRl of the camera rotation axes can be computed using Equation (26):

(26) TRrRl=0.930970.012249−0.3656522.627−0.0060964−0.999950.0234870.78731−0.36537−0.02162−0.9306537.120001

After obtaining the initial pose matrices $T_{r}$, $T_{l}$, and the relative pose matrix TRrRl of the camera rotation axes, we can calculate the relative pose matrices of the left and right cameras at different angles using Equations (2)–(10). To visually demonstrate the accuracy of the algorithm, we conducted stereo calibration at various angles to obtain their accurate relative pose matrices. These results were then compared with the calculated relative pose matrices to derive the computation errors, as shown in Table 3:

The error can be obtained by subtracting the calibration results from the computed results, as shown in Table 4:

Through error analysis, it can be calculated that the average computation error of the proposed algorithm for the relative rotation matrix of the left and right cameras at various angles is 0.19%, while the average computation error for the relative translation vector is 3.06%. These results indicate that the algorithm demonstrates a high level of accuracy in calculating the extrinsic parameters of the stereo camera system.

## 5. Conclusions

This paper proposes a method to calibrate static parameters and update extrinsic parameters dynamically for active stereo vision systems, and we designed a two-DOF bionic active camera system to verify the algorithm. This system allowed for the dynamic adjustment of the FOV, mimicking the motion of certain fish eyes. By establishing a rotation axis coordinate system and a camera coordinate system, the relative relationship between the active camera and the other cameras is transformed into a relationship calculation between the active camera and a fixed coordinate system. Then, dynamic pose matrix updates can be calculated to derive the extrinsic parameters of the stereo camera at various angles. An experimental platform is constructed based on the camera, and experiments validate the feasibility and accuracy of the dynamic extrinsic parameters’ updating method. This capability enhanced the visual perception range and precision, and provides new ideas for the application of cameras in the field of robotics. In the future, we will continue to develop applications such as robot navigation based on this.

## Figures and Tables

**Table 1:** Some parameters of the camera.

Performance Indicator	Parameter
Pixel	8 million
Resolution	1920 × 1080
Horizontal FOV	$145^{\circ }$–$225^{\circ }$
Vertical FOV	−$70^{\circ }$–$70^{\circ }$
Video frame rate	30 fps
Power consumption	≤15 W

**Table 2:** Translation vector relative to the initial position.

Angle θ	TiCl	TiCr
$5^{\circ }$	$$\left[\begin{array}{cccc} 0.9952 & 0.00057411 & 0.09783 & 5.1718\\ -0.00027688 & 1 & -0.0030518 & 2.1846\\ -0.097831 & 0.0030101 & 0.9952 & -1.2521\\ 0 & 0 & 0 & 1 \end{array}\right]$$	$$\left[\begin{array}{cccc} 0.99504 & -0.00085912 & -0.099446 & -4.0205\\ 0.00075176 & 1 & -0.0011171 & 0.5033\\ 0.099446 & 0.0010368 & 0.99504 & 0.10301\\ 0 & 0 & 0 & 1 \end{array}\right]$$
$10^{\circ }$	$$\left[\begin{array}{cccc} 0.97901 & 0.0015026 & 0.20381 & 6.2427\\ -0.0012125 & 1 & -0.0015479 & 0.55918\\ -0.20381 & 0.0012683 & 0.97901 & -2.2025\\ 0 & 0 & 0 & 1 \end{array}\right]$$	$$\left[\begin{array}{cccc} 0.97962 & -0.00059376 & -0.20086 & -7.7721\\ 0.00031852 & 1 & -0.0014026 & 0.39557\\ 0.20087 & 0.00131 & 0.97962 & -0.56915\\ 0 & 0 & 0 & 1 \end{array}\right]$$
$15^{\circ }$	$$\left[\begin{array}{cccc} 0.9523 & 0.0027534 & 0.30515 & 6.8178\\ -0.0020205 & 0.99999 & -0.0027175 & 1.2785\\ -0.30516 & 0.0019713 & 0.9523 & -3.1287\\ 0 & 0 & 0 & 1 \end{array}\right]$$	$$\left[\begin{array}{cccc} 0.95143 & -0.0019584 & -0.30787 & -10.414\\ 0.0018851 & 1 & -0.00053533 & -0.97069\\ 0.30787 & -7.1047\times 10^{-5} & 0.95143 & -1.8351\\ 0 & 0 & 0 & 1 \end{array}\right]$$
$20^{\circ }$	$$\left[\begin{array}{cccc} 0.9118 & 0.0045513 & 0.4106 & 11.077\\ -0.0035028 & 0.99999 & -0.003306 & 1.5236\\ -0.41061 & 0.0015761 & 0.91181 & -5.9663\\ 0 & 0 & 0 & 1 \end{array}\right]$$	$$\left[\begin{array}{cccc} 0.91083 & -0.0028104 & -0.41278 & -14.814\\ 0.0025537 & 1 & -0.0011736 & -0.63481\\ 0.41278 & 1.487\times 10^{-5} & 0.91083 & -3.8462\\ 0 & 0 & 0 & 1 \end{array}\right]$$

**Table 3:** Relative pose matrices for different angles.

Angle θ	TCrCl(θ)calibrated	TCrCl(θ)calculated
$0^{\circ }$	$$\left[\begin{array}{cccc} 0.9952 & -0.0091124 & -0.097401 & -53.228\\ 0.0076324 & 0.99985 & -0.015556 & 1.6822\\ 0.097528 & 0.014738 & 0.99512 & -4.0524\\ 0 & 0 & 0 & 1 \end{array}\right]$$	$$\left[\begin{array}{cccc} 0.9952 & -0.0091124 & -0.097401 & -53.228\\ 0.0076324 & 0.99985 & -0.015556 & 1.6822\\ 0.097528 & 0.014738 & 0.99512 & -4.0524\\ 0 & 0 & 0 & 1 \end{array}\right]$$
$5^{\circ }$	$$\left[\begin{array}{cccc} 0.95669 & -0.011066 & -0.29089 & -61.871\\ 0.0071682 & 0.99987 & -0.01446 & -0.089517\\ 0.29101 & 0.011749 & 0.95665 & -9.5537\\ 0 & 0 & 0 & 1 \end{array}\right]$$	$$\left[\begin{array}{cccc} 0.95665 & -0.011895 & -0.29091 & -58.857\\ 0.0062587 & 0.99989 & -0.015055 & 1.733\\ 0.29105 & 0.013171 & 0.95662 & -9.5486\\ 0 & 0 & 0 & 1 \end{array}\right]$$
$10^{\circ }$	$$\left[\begin{array}{cccc} 0.87507 & -0.013182 & -0.48382 & -65.623\\ 0.0056896 & 0.99984 & -0.016951 & 1.4345\\ 0.48396 & 0.01208 & 0.875 & -16.329\\ 0 & 0 & 0 & 1 \end{array}\right]$$	$$\left[\begin{array}{cccc} 0.875 & -0.014717 & -0.48379 & -63.875\\ 0.0051925 & 0.99994 & -0.014147 & 1.7939\\ 0.48394 & 0.011023 & 0.87504 & -16.808\\ 0 & 0 & 0 & 1 \end{array}\right]$$
$15^{\circ }$	$$\left[\begin{array}{cccc} 0.75129 & -0.015933 & -0.65978 & -66.978\\ 0.0067925 & 0.99984 & -0.016411 & -0.76268\\ 0.65993 & 0.007848 & 0.75128 & -24.237\\ 0 & 0 & 0 & 1 \end{array}\right]$$	$$\left[\begin{array}{cccc} 0.7512 & -0.017278 & -0.65974 & -67.879\\ 0.0045907 & 0.99999 & -0.013145 & 1.8493\\ 0.65991 & 0.0085006 & 0.75131 & -25.273\\ 0 & 0 & 0 & 1 \end{array}\right]$$
$20^{\circ }$	$$\left[\begin{array}{cccc} 0.58463 & -0.018576 & -0.81109 & -72.913\\ 0.0067535 & 0.99981 & -0.018031 & -0.78951\\ 0.81127 & 0.0050637 & 0.58465 & -35.014\\ 0 & 0 & 0 & 1 \end{array}\right]$$	$$\left[\begin{array}{cccc} 0.58452 & -0.019633 & -0.81103 & -70.347\\ 0.004516 & 1 & -0.011953 & 1.9038\\ 0.81123 & 0.0053703 & 0.5847 & -35.292\\ 0 & 0 & 0 & 1 \end{array}\right]$$

**Table 4:** Error for different angles.

Angle θ	$0^{\circ }$	$5^{\circ }$	$10^{\circ }$	$15^{\circ }$	$20^{\circ }$	Average
Rotation Error (%)	0.00	0.11	0.20	0.24	0.38	0.19
Translation Error (%)	0.00	5.90	2.80	4.07	4.74	3.06

## Data Availability

The data generated during the current study are available from the corresponding author on reasonable request.
